# Supplementary material for: Genetic Insights into the Extremely Dwarf Hibiscus syriacus var. micranthus: Complete Chloroplast Genome Analysis and Development of a Novel dCAPS Marker
Source: Curr Issues Mol Biol. 2024 Mar 21;46(3):2757–71. doi: 10.3390/cimb46030173 (PMC10968724; doi:10.3390/cimb46030173)
Supplement: Supplementary file 1 [file cimb-46-00173-s001.zip › cimb-2888908-supplementary.pdf]

Table S1. Individual-specific variant distribution (unit: bp).

Name	Number of substitutions	Number of insertions	Number of deletions	Number of total variants
HS N.M.520	2	5	10	17
HSVM	1	3	0	4
HS N.M.521	0	1	0	1
HS 'Tamra'	44	150	268	462
